# Serum creatinine/cystatin C ratio as a case-finding tool for low handgrip strength in Chinese middle-aged and older adults

**DOI:** 10.1038/s41598-020-71028-4

**Published:** 2020-08-20

**Authors:** Lingling Tan, Ruicen Li, Xiaoyi Hu, Yuan Zhu, Ting Bao, Yun Zuo, Ming Yang

**Affiliations:** 1 Center of Gerontology and Geriatrics, West China Hospital, Sichuan University, No. 37 Guoxue Lane, Chengdu, China; 2 Health Management Center, West China Hospital, Sichuan University, No. 37 Guoxue Lane, Chengdu, China; 3 Health Management Center, Shangjin Nanfu Hospital, Chengdu, China; 4 Precision Medicine Research Center, West China Hospital, Sichuan University, No. 37 Guoxue Lane, Chengdu, China

**Keywords:** Biomarkers, Diagnostic markers, Biomarkers, Risk factors

## Abstract

Measuring handgrip strength is the initial step to diagnose sarcopenia. To investigate whether the serum creatinine (Cr)/cystatin C (CysC) ratio could serve as a case-finding tool for low handgrip strength, we conducted a diagnostic accuracy study. Adults (aged ≥ 40 years) with normal renal function were recruited. Trained nurses collected blood samples and conducted the anthropometric measurements and handgrip strength test. The serum concentrations of Cr, CysC, and other biomarkers were measured. We recruited 1098 men and 1241 women. The Cr/CysC ratio was significantly associated with AWGS-defined low handgrip strength among men and women. The areas under the receiver operating characteristic curves were 0.79 among men and 0.78 among women for using the Cr/CysC ratio to identify AWGS-defined low handgrip strength. We set the Cr/CysC ratio cut-off values at < 8.9 among men and < 8.0 among women. The corresponding sensitivity values were 64.9% among men and 63.1% among women, while the specificity values were 83.7% among men and 77.5% among women. In conclusion, the Cr/CysC ratio is positively and linearly associated with handgrip strength and may be helpful for screening low handgrip strength in Chinese middle-aged and older adults dwelling in communities.

## Introduction

Sarcopenia is a skeletal muscle disease characterized by loss of muscle mass and muscle function^1^. This disease is prevalent worldwide, especially among older adults, and has become an important public health issue^1,2^. The European Working Group on Sarcopenia in Older Adults (EWGSOP) criteria are the most widely used criteria for managing sarcopenia^3^. The updated recommendations of the EWGSOP (EWGSOP2) indicate that the diagnosis of sarcopenia depends on the measurement of muscle strength (generally handgrip strength measured using a dynamometer) and the measurement of muscle mass using dual-energy X-ray (DXA), computed tomography (CT), and magnetic resonance imaging (MRI)^4^.

According to the EWGSOP2^4^, low handgrip strength is the prerequisite for diagnosing sarcopenia. Furthermore, low handgrip strength per se is associated with a range of adverse health outcomes in different populations, such as depression^5^, poor quality of life^6^, and mortality^7^. Therefore, it is important to screen low handgrip strength in older adults. However, the measurement of handgrip strength needs different types of dynamometers, such as Jamar dynamometer, which are not routinely accessible in clinical practice, especially in primary care. Therefore, a serum biomarker based on routine blood test results would be useful for predicting or identifying low handgrip strength, which could then be used in screening sarcopenia and predicting the risk of adverse health outcomes in broader populations^8^.

Serum creatinine (Cr) is a well-known biomarker of renal function and muscle mass, as 90% of its precursor (creatine phosphate) is produced in muscle and its excretion depends on the kidneys^9^. Serum cystatin C (CysC) is a small protein that is excreted by all nucleated cells and is commonly used in clinical practice to assess renal function^10^. To compromise the influence of renal function to serum Cr concentrations, Kashani et al. recently developed a new biomarker for estimating muscle mass, which they named the “sarcopenia index” (serum Cr [mg/dL] /serum CysC [mg/L] × 100)^11^. Our team found that the sarcopenia index could not accurately identify low skeletal muscle mass or sarcopenia in community-dwelling older adults^12^; however, other studies indicated that the Cr/CysC ratio was a marker of skeletal muscle mass in different populations^13,14^.

Moreover, we recently found that the sarcopenia index was correlated with handgrip strength (r^2^ = 0.24, *p* < 0.001) among older Chinese inpatients^15^. A more recent report has indicated that handgrip strength in 677 community-dwelling older Japanese adults was correlated with the Cr/CysC ratio^16^. These findings imply that the Cr/CysC ratio may help identify low handgrip strength. This study aimed to determine whether the Cr/CysC ratio was correlated with handgrip strength in a large Chinese population with a broader age range, and to identify a suitable diagnostic cut-off value for the Cr/CysC ratio.

## Methods

### Participants

This cross-sectional population-based study was conducted in Chengdu, China between January and October 2019. According to previous studies^17,18^, the highest handgrip strength was observed in the 4th decade of life (31–40 years) in different populations; therefore, we invited adults who were ≥ 40 years old and living in their community to participate in this study. The exclusion criteria were renal function impairment (an estimated glomerular filtration rate of < 60 mL/min/1.73 m^2^), any hand and arm injuries or disorders that might influence the measurement of handgrip strength, and any type of cancer. Trained nurses collected the participants’ clinical information and blood samples, and performed the anthropometric measurements and handgrip strength test. The study protocol was approved by the Biomedical Ethics Committee of West China Hospital of Sichuan University, and all participants provided written informed consent. All the methods in this study were in accordance with the relevant guidelines and regulations.

### Measurement of handgrip strength

Handgrip strength was measured by training nurses using a digital grip dynamometer (EH101, Xiangshan Inc., Guangdong, China), which has an adjustable grip span and provides measurements of 0.1–100.0 kg in 0.1-kg increments. The recommendations from the Chinese National Physical Fitness Evaluation Standard^19^ were used for the measurement protocol. First, the participants received instructions regarding how to use the dynamometer. Second, they were asked to stand with their feet shoulder-width apart and with their elbows fully extended. Third, they were asked to hold the dynamometer in a neutral position with 90° of flexion at the index finger, and without the dynamometer touching their bodies. Fourth, they were asked to squeeze the grip continuously with full force for ≥ 3 s. No verbal encouragement or visual feedback was allowed during the measurement process. The participants performed three alternating trials for each hand, with rest periods of ≥ 30 s between each measurement. The handgrip strength result was defined as the maximum value from the six measurements.

### Measurement of clinical and laboratory parameters

The following clinical characteristics were collected via face-to-face interviews: age, sex, smoking status, alcohol drinking status, education, and histories of hypertension and diabetes. Height was measured to the nearest 0.5 cm and weight was measured to the nearest 0.1 kg using an automatic device (Ultrasonic body scale; SONKA Inc., Shenzhen, China). Body mass index (BMI) was calculated as weight/height^2^ (kg/m^2^).

Blood samples were obtained from the antecubital vein of each participant during the morning after ≥ 8 h of fasting. Serum concentrations of uric acid, Cr, CysC, creatine kinase (CK), and albumin were detected using the Cobas c702 chemistry autoanalyzer (Roche Diagnostics, Switzerland). Serum C-reactive protein (CRP) concentrations were detected using an immunoturbidimetric technique with the Modular Analytics Cobas 6000 analyzer (Roche Diagnostics, Switzerland). Hemoglobin concentrations were detected using a Sysmex XE-500 analyzer (Sysmex Corporation, Japan). The Cr/CysC ratio was calculated as Cr/CysC ratio = serum Cr (mg/dL)/serum CysC (mg/L) × 10.

### Statistical analysis

Continuous data were presented as mean and standard deviation (SD) or median and interquartile range (IQR) as appropriate, while categorical data were presented as number and percentage. Inter-group differences were evaluated using one-way analysis of variance for normally distributed variables, using the Mann–Whitney U test for non-normally distributed variables, and using the chi-squared test for categorical variables. The handgrip strength analyses were stratified according to sex because previous studies reported the significant difference between men and women^4–6^.

Pearson’s correlation coefficient or Spearman’s correlation coefficient was used as appropriate to evaluate the relationships between handgrip strength and the Cr/CysC ratio, age, CK concentration, and other serum biomarkers. Multiple linear regression analysis was also performed to explore the linear relationships between handgrip strength and these biomarkers. Logistic regression analysis with backward selection was performed to explore the biomarkers associated with low handgrip strength, which was identified based on the EWGSOP2 criteria (< 27 kg for men and < 16 kg for women)^4^ and the Asian Working Group for Sarcopenia criteria (AWGS, < 26 kg for men and < 18 kg for women)^20^.

Receiver operating characteristic (ROC) curve analysis was used to evaluate the utility of the Cr/CysC ratio for identifying low handgrip strength, based on the area under the ROC curve (AUC) and 95% confidence interval (CI). The Youden index (sensitivity + specificity – 1) was calculated to determine the optimal cutoff points for identifying low handgrip strength among men and women^21^. The values for sensitivity, specificity, the positive likelihood ratio, and the negative likelihood ratio were also calculated. All statistical analyses were performed using IBM SPSS software (version 26.0; IBM Corp., Armonk, NY, US) and MedCalc Statistical Software (version 15.2; MedCalc Software bvba, Ostend, Belgium). All statistical tests were two-sided and statistical significance was identified based on a P-value of < 0.05.

## Results

### Study population

We recruited 2539 adults to participate in this study, although some participants were excluded for the following reasons: estimated glomerular filtration rate of < 60 mL/min/1.73 m^2^ (n = 103), any type of cancer (n = 65), hand and arm injuries or disorders (n = 32), and missing data regarding handgrip strength (n = 29). Thus, 2339 participants (median age: 55 years, range 40–89 years) were considered eligible for the analyses, including 1098 men and 1241 women (Table 1).

**Table 1:** Subject characteristics according to sex.

	Total (n = 2339)	Sex		
		Men (n = 1098)	Women (n = 1241)	*p *value
Age (years)*	55 (17)	54 (17)	55 (17)	0.362
Current smokers, n (%)	528 (22.6)	514 (46.8)	14 (1.1)	< 0.001
Current alcohol drinkers, n (%)	729 (31.2)	640 (58.3)	89 (7.2)	< 0.001
Hypertension, n (%)	381 (18.3)	205 (21.6)	156 (15.3)	< 0.001
Diabetes, n (%)	142 (6.1)	85 (7.8)	56 (4.5)	0.001
Body mass index (kg/m^2^)^†^	23.6 (3.0)	24.4 (2.9)	23.0 (3.0)	< 0.001
Handgrip strength (kg)	28.2 (16.1)*	38.5 (7.8)^†^	23.1 (5.1)^†^	< 0.001
C-reactive protein (mg/L)*	2.1 (1.5)	2.1 (1.5)	2.1 (1.5)	0.421
Creatine kinase (IU/L)*	94.0 (54.0)	104.0 (57.8)	84.0 (47.8)	< 0.001
Uric acid (μmol/L)^†^	333.3 (83.6)	377.5 (76.9)	294.1 (68.3)	< 0.001
Creatinine (mg/dL)*	0.8 (0.2)	0.9 (0.2)	0.7 (0.1)	< 0.001
Cystatin C (mg/L)*	0.8 (0.1)	0.9 (0.1)	0.8 (0.2)	< 0.001
Cr/CysC ratio^†^	9.7 (1.8)	10.5 (1.7)	8.9 (1.5)	< 0.001
Hemoglobin (g/L)^†^	144.5 (16.4)	156.4 (12.5)	134.2 (11.8)	< 0.001
Albumin (g/L)^†^	47.1 (3.0)	47.6 (3.0)	46.7 (3.0)	< 0.001

*Data are presented as median (interquartile range).

^†^Data are presented as mean (standard deviation).

To detect the difference between men and women, one-way ANOVA was used for the continuous variables with normal distribution; the Mann–Whitney U test was used for the continuous variables with non-normal distribution; the chi-squared test was used for the categorical variables.

Cr/CysC ratio = serum creatinine (mg/dL)/serum cystatin C (mg/L) × 10.

### Factors associated with handgrip strength

Supplementary Table 1 shows the simple correlations between handgrip strength and potential covariates. Among both men and women, handgrip strength negatively correlated with age (men: r = − 0.329, *p* < 0.001; women: r = − 0.341, *p* < 0.001), CRP concentration (men: r = − 0.043, *p* = 0.020; women: r = − 0.072, *p* = 0.004), and CysC concentration (men: r = − 0.207, *p* < 0.001; women: r = − 0.276, *p* < 0.001), while handgrip strength positively correlated with the Cr/CysC ratio (men: r = 0.376, *p* < 0.001; women: r = 0.407, *p* < 0.001), hemoglobin concentration (men: r = 0.304, *p* < 0.001; women: r = 0.157, *p* < 0.001), and albumin concentration (men: r = 0.414, *p* < 0.001; women: r = 0.341, *p* < 0.001). Among men, but not among women, BMI (r = 0.134, *p* < 0.001) and CK concentration (r = 0.008, *p* = 0.006) positively correlated with handgrip strength among men. Uric acid concentration positively correlated with handgrip strength among men (r = 0.083, *p* = 0.006), but negatively correlated with handgrip strength among women (r = − 0.118, *p* = 0.001).

**Figure 1:**
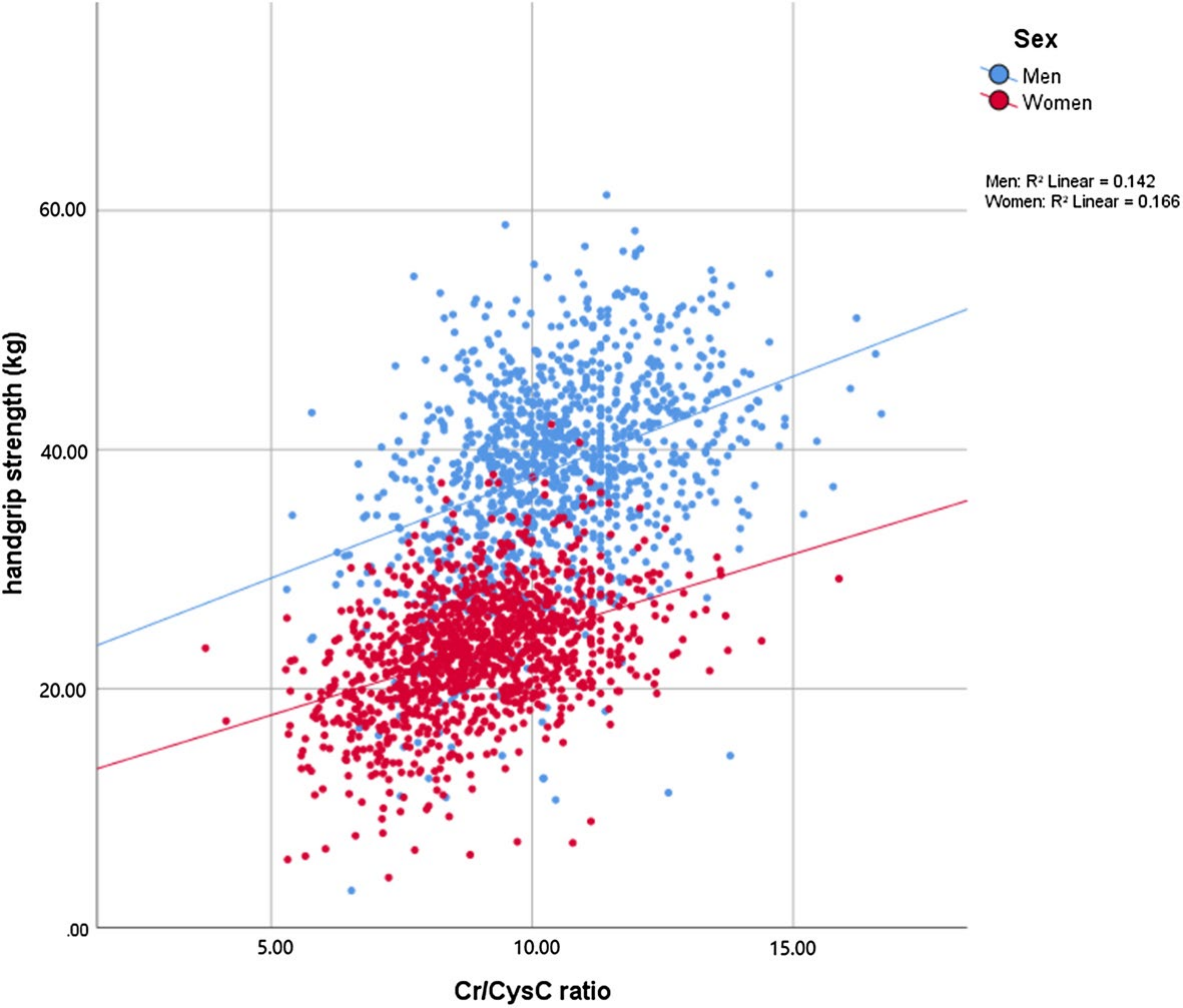
The linear correlation between handgrip strength and the creatinine/cystatin C ratio. *CR* creatinine, *CysC* cystatin C.

Figure 1 shows the positive linear correlations between handgrip strength and the Cr/CysC ratio among men (r = 0.376, *p* < 0.001) and among women (r = 0.407, *p* < 0.001). In addition, as shown in Supplementary Fig. 1, the Cr/CysC ratio was negatively correlated with age among men (r = − 0.467, *p* < 0.001) and among women (r = − 0.587, *p* < 0.001). Similarly, handgrip strength negatively correlated with age in both men (r = − 0.537, *p* < 0.001) and women (r = − 0.485, *p* < 0.001).

Supplementary Table 2 shows the multiple linear regression analysis of handgrip strength and potential biomarkers. Among both men and women, age was negatively associated with handgrip strength (men: β = − 0.261, *p* < 0.001; women: β = − 0.302, *p* < 0.001), whereas the Cr/CysC ratio was positively associated with handgrip strength (men: β = 0.112, *p* = 0.013; women: β = 0.170, *p* = 0.001). Uric acid was negatively associated with handgrip strength among women (β = − 0.110, *p* = 0.011), although this association was not significant among men (β = − 0.029, *p* = 0.515).

**Table 2:** Factors associated with low handgrip strength defined using the EWGSOP2 or AWGS recommendations according to logistic regression with backward selection.

	Coefficient	SE	Wald	*p* value	OR (95% CI)
**EWGSOP2**					
*Men*					
Age (per year)	0.148	0.017	75.590	< 0.001	1.16 (1.12–1.20)
Cr/CysC ratio (per 1 SD)	− 0.455	0.181	6.348	0.012	0.64 (0.45–0.90)
*Women*					
Age (per year)	0.141	0.015	87.629	< 0.001	1.15 (1.12–1.29)
Cr/CysC ratio (per 1 SD)	− 0.566	0.181	9.804	0.002	0.57 (0.40–0.81)
**AWGS**					
*Men*					
Age (per year)	0.164	0.020	68.898	< 0.001	1.18 (1.13–1.22)
Cr/CysC ratio (per 1 SD)	− 0.464	0.204	5.155	0.023	0.63 (0.42–0.94)
*Women*					
Age (per year)	0.121	0.011	111.072	0.000	1.13 (1.10–1.15)
Cr/CysC ratio (per 1 SD)	− 0.659	0.144	20.986	0.000	0.52 (0.39–0.69)

AWGS, Asian Working Group for Sarcopenia; CI, confidence interval, EWGSOP2, the updated recommendations of the European Working Group on Sarcopenia in Older Adults; OR, odds ratio, SD, standard deviation; SE, standard error.

Cr/CysC ratio = serum creatinine (mg/dL)/serum cystatin C (mg/L) × 10.

### Using the Cr/CysC ratio to identify low handgrip strength

Table 2 shows the factors associated with AWGS-defined or EWGSOP2-defined low handgrip strength according to the logistic regression analysis. The Cr/CysC ratio was significantly associated with AWGS-defined low handgrip strength among both men and women (odds ratio [OR] per 1 SD among men: 0.63, 95% CI 0.42–0.94; OR per 1 SD among women: 0.52, 95% CI 0.39–0.69). Similar results were observed using the EWGSOP2 criteria (Table 2).

**Figure 2:**
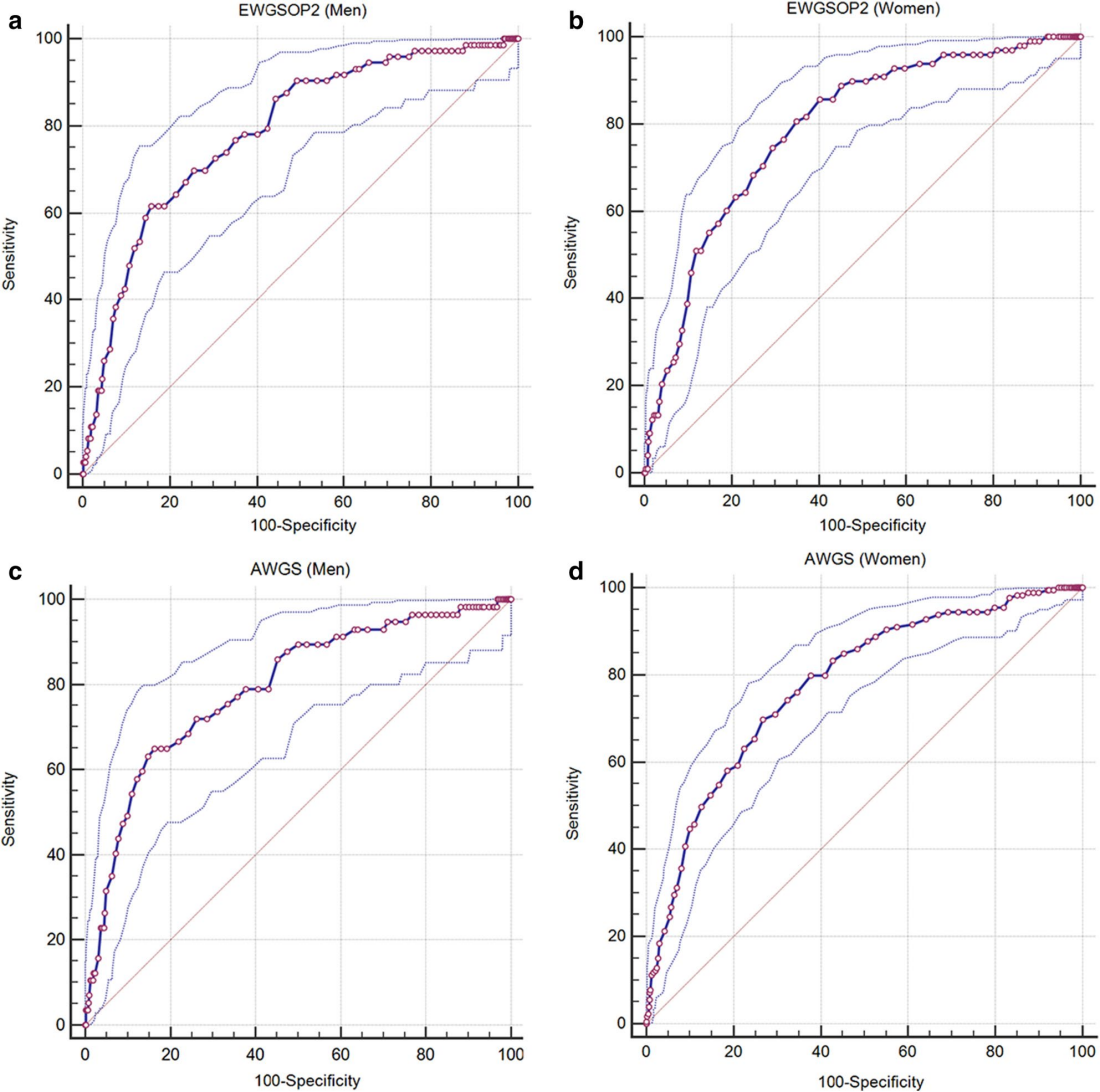
Receiver operating characteristic curves for the creatinine/cystatin C ratio and low handgrip strength defined using the EWGSOP2 ((**a**) men, (**b**) women) and AWGS ((**c**) men, (**d**) women). CR, creatinine; CysC, cystatin C, EWGSOP2: the updated recommendations of the European Working Group on Sarcopenia in Older Adults; AWGS, Asian Working Group for Sarcopenia.

Table 3 shows the results of the sensitivity specificity analyses for using the Cr/CysC ratio to identify AWGS-defined or EWGSOP2-defined low handgrip strength. Using the AWGS criteria as the reference standard, the AUC for using the Cr/CysC ratio to identify low handgrip strength was 0.79 among men (95% CI 0.77–0.82) and 0.78 among women (95% CI 0.76–0.80). Similar results were observed using the EWGSOP2 criteria as the reference standard (Table 3). Based on the Youden index method, we set the Cr/CysC ratio cut-off values for identifying AWGS-defined low handgrip strength as < 8.9 among men and < 8.0 among women. Among men the sensitivity value was 64.9% (95% CI 51.1–77.1%) and the specificity value was 83.7% (95% CI 81.3–85.9%), while among women the sensitivity value was 63.1% (95% CI 55.6–70.2%) and the specificity value was 77.5% (95% CI 74.9–80.0%). Similar results were observed using the EWGSOP2 criteria (Table 3). Figure 2 shows the ROC curves for the Cr/CysC ratio and different reference standards among men and women.

**Table 3:** Sensitivity and specificity for using the Cr/CysC ratio to predict different definitions of low handgrip strength.

	AUC	Cut-off for the Cr/CysC ratio	Sensitivity (%)	Specificity (%)	+ LR	-LR
**EWGSOP2**						
Men	0.79 (0.76–0.81)	8.9	61.6 (49.5–72.8)	84.2 (81.8–86.4)	3.9 (3.1–4.9)	0.5 (0.3–0.6)
Women	0.79 (0.77–0.81)	8.0	68.4 (58.2–77.4)	75.1 (72.5–77.6)	2.8 (2.3–3.2)	0.4 (0.3–0.6)
**AWGS**						
Men	0.79 (0.77–0.82)	8.9	64.9 (51.1–77.1)	83.7 (81.3–85.9)	4.0 (3.1–5.0)	0.4 (0.3–0.6)
Women	0.78 (0.76–0.80)	8.0	63.1 (55.6–70.2)	77.5 (74.9–80.0)	2.8 (2.4–3.3)	0.5 (0.4–0.6)

Data are presented with the 95% confidence interval in parentheses.

AWGS, Asian Working Group for Sarcopenia; AUC, area under the curve; EWGSOP2, the updated recommendations of the European Working Group on Sarcopenia in Older People; + LR, positive likelihood ratio; -LR, negative likelihood ratio.

## Discussion

Based on a large population-based cohort, our study revealed that the Cr/CysC ratio was positively and linearly associated with handgrip strength among Chinese adults who were ≥ 40 years old. The Cr/CysC ratio also appears to be a useful biomarker for identifying low handgrip strength (AUC of approximately 0.8 among both men and women), with Cr/CysC ratio cut-off values set at < 8.9 for men and < 8.0 for women. These cut-off values provided a sensitivity of > 60% and a specificity of > 80% among men, as well as a sensitivity of > 60% and a specificity of approximately 75% among women. Given that Cr and CysC concentrations are commonly tested in clinical practice and routine health check-ups, the Cr/CysC ratio may help identify patients with low muscle strength in clinical settings.

The Cr/CysC ratio and the “sarcopenia index” are very similar markers that are based on serum Cr and CysC concentrations, with the “sarcopenia index” being equal to the Cr/CysC ratio × 10. However, it is noteworthy that there are different definitions for “sarcopenia index,” which are based on various serum biomarkers. For example, Harada et al.^22^ defined their “sarcopenia index” based on serum adiponectin and sialic acid concentrations, instead of serum Cr and CysC concentrations. Therefore, to avoid confusion, we used the term “Cr/CysC ratio” to differentiate our calculation from the “sarcopenia index.”

Our finding that the Cr/CysC ratio was positively associated with handgrip strength agrees with the results from two recent studies involving different populations. Kusunoki et al.^16^ reported that the Cr/CysC ratio was positively correlated with handgrip strength (r = 0.59, *p* < 0.001) and knee extension muscle strength (r = 0.49, *p* < 0.001) among 677 Japanese community-dwelling older adults. Tabara et al.^23^ also reported that the Cr/CysC ratio was independently associated with handgrip strength, regardless of muscle mass, among 1329 Japanese community-dwelling older adults.

In this context, low handgrip strength is a key component of sarcopenia^4^ and a predictor of various adverse health outcomes^5–7,24,25^. Therefore, it would be valuable to identify persons with low handgrip strength in both research studies and clinical practice. To the best of our knowledge, ours is the first study to evaluate the predictive value of the Cr/CysC ratio for identifying low handgrip strength in a large population. Serum Cr and CysC concentrations are routinely tested in clinical practice, and this testing provides high reproducibility at low costs^10,26^. Therefore, the Cr/CysC ratio may be useful as a marker during screening for low handgrip strength in both research and clinical settings.

Because the measurement of handgrip strength is recommended as the initial step in a sarcopenia assessment^4^, our findings also imply that the Cr/CysC ratio might be useful in sarcopenia screening. Several previous studies have recently addressed this issue, although the results remain controversial. For example, Kusunoki et al.^16^ performed a cross-sectional study that revealed the Cr/CysC ratio was associated with sarcopenia among Japanese community-dwelling older adults. In addition, Osaka et al.^27^ argued that the Cr/CysC ratio was a useful biomarker for sarcopenia screening based on data from 285 Japanese patients with type 2 diabetes. However, Singhal et al. ^28^ reported that the Cr/CysC ratio was not associated with sarcopenia among 100 older outpatients, from a tertiary hospital in India. Our team has also previously found that the “sarcopenia index” (i.e., the Cr/CysC ratio × 10) could not accurately identify sarcopenia among community-dwelling older Chinese adults^12^. Therefore, further large population-based studies are needed to clarify the potential role of the Cr/CysC ratio in sarcopenia screening.

The present study has several limitations. First, the cross-sectional design precludes a conclusion regarding the causality of the relationship between the Cr/CysC ratio and handgrip strength. Second, because we did not collect data regarding muscle mass, we could not directly analyze the association between the Cr/CysC ratio and skeletal muscle mass or sarcopenia. Third, serum Cr and CysC concentrations are significantly influenced by renal function. This suggests that the Cr/CysC ratio may not be suitable for identifying low muscle strength in patients with abnormal renal function, and sarcopenia has a relatively high prevalence among patients with chronic kidney diseases^29^. Fourth, our study evaluated Chinese adults and previous studies have revealed ethnicity-related variations in handgrip strength^30,31^, which suggests that caution is needed when extending our findings to other ethnic populations. Last, we did not assess the Shrunken Pore Syndrome (SPS), a new syndrome that may display a low Cr/CysC ratio even when renal function is normal and no sarcopenia is present^32,33^. The prevalence of SPS is unknown in community-dwelling older adults; however, the prevalence of SPS was only 2.1% in patients undergoing elective coronary artery bypass grafting^34^.

## Conclusion

The Cr/CysC ratio was positively and linearly associated with handgrip strength among a population-based sample of adults who were ≥ 40 years old and had normal renal function. Cr and CysC concentrations are routinely tested in different clinical settings including health check-ups. In China, community-dwelling older adults have an opportunity to receive a yearly check-up (including the test of Cr and CysC) without charge. Therefore, the Cr/CysC ratio may be helpful to detect low muscle strength in this population without adding any time-consuming steps, medical device, or financial burden.

Most recently, the updated version of the AWGS (AWGS 2019) has defined a new entity of “possible sarcopenia” that is low handgrip strength or low physical performance^35^. The Cr/CysC ratio may also be used as a “case-finding” tool of “possible sarcopenia” (like the SARC-F questionnaire) in persons who received health check-ups or routine tests for other reasons. Based on our results, we suggest using Cr/CysC ratio cut-offs of < 8.9 among men and < 8.0 among women, as they provided acceptable sensitivity and modest-to-high specificity for identifying low handgrip strength in Chinese community-dwelling adults with normal renal function. Nevertheless, these cut-offs may not be suitable for all ethnic populations, and further large studies are needed to validate their utility in other ethnic populations.

## Supplementary information

Supplementary Information.

## Data Availability

The datasets generated during and/or analyzed during the current study are available from the corresponding author on reasonable request.
