# Supplementary material for: Serum creatinine/cystatin C ratio as a case-finding tool for low handgrip strength in Chinese middle-aged and older adults
Source: Sci Rep. 2020 Aug 20;10:14028. doi: 10.1038/s41598-020-71028-4 (PMC7441391; doi:10.1038/s41598-020-71028-4)
Supplement: Supplementary file 1 — Supplementary Information. [file 41598_2020_71028_MOESM1_ESM.pdf]

Title Page

Title:

Serum creatinine/cystatin C ratio as a case-finding tool for low handgrip strength in Chinese middle-aged and older adults

Authors:

Lingling Tan, MS [1], Ruicen Li, MS [2], Xiaoyi Hu, MSN [1,3], Yuan Zhu, MD[2], Ting Bao, MD[2], Yun Zuo, BSN[3], Ming Yang, MD[1,4]

Affiliations and addresses of all authors:

1. Center of Gerontology and Geriatrics, West China Hospital, Sichuan University, No. 37 Guoxue Lane, Chengdu, China
2. Health Management Center, West China Hospital, Sichuan University, No. 37 Guoxue Lane, Chengdu, China
3. Health Management Center, Shangjin Nanfu Hospital, Chengdu, China.
4. Precision Medicine Research Center, West China Hospital, Sichuan University, No. 37 Guoxue Lane, Chengdu, China

Corresponding author:

Ming Yang, MD

Phone: +86 28 8542 2321

Fax: +86 28 85542 2321

Email: yangmier@gmail.com

Supplementary Figure 1. The linear correlations between age and handgrip strength (a) or the creatinine/cystatin C ratio (b). CR: creatinine; CysC: cystatin C.

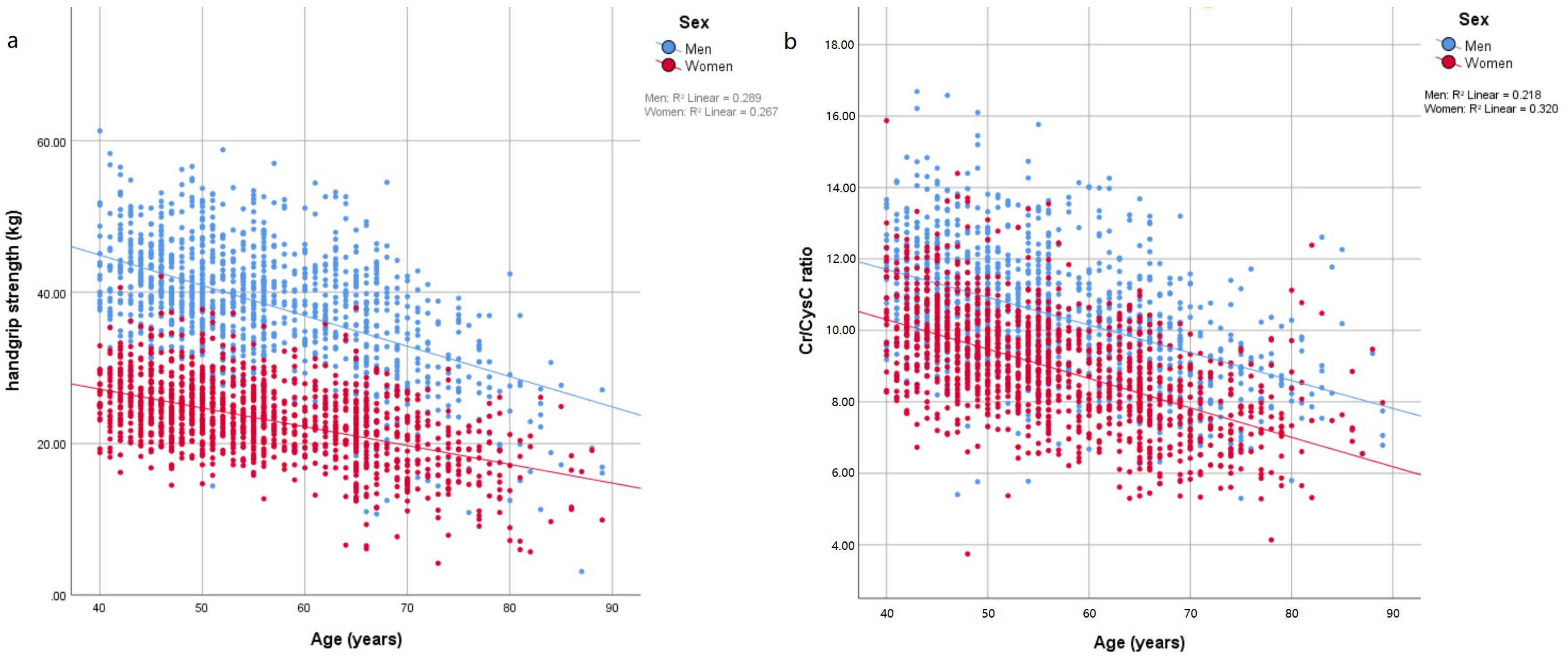

Supplementary Table 1. Correlations between handgrip strength and individual factors

	Men (n=1,098)		Women (n=1,241)	
	r	P-value	r	P-value
Age (years)*	–0.329	<0.001	–0.341	<0.001
Body mass index (kg/m^2) †	0.134	<0.001	–0.041	0.145
C-reactive protein (mg/L) *	–0.043	0.020	–0.072	0.004
Creatine kinase (IU/L) *	0.080	<0.001	0.004	0.847
Uric acid (µmol/L) †	0.083	0.006	–0.118	0.001
Creatinine (mg/dL) *	0.036	0.078	–0.042	0.080
Cystatin C (mg/L) *	–0.207	<0.001	–0.276	<0.001
Cr/CysC ratio†	0.376	<0.001	0.407	<0.001
Hemoglobin (g/L) †	0.304	<0.001	0.157	<0.001
Albumin (g/L) †	0.414	<0.001	0.341	<0.001

* Spearman's correlation coefficient was used because of the data's non-normal distribution.

† Pearson's correlation coefficient was used because of the data's normal distribution.

Cr/CysC ratio = serum creatinine (mg/dL) / serum cystatin C (mg/L) × 10

Supplementary Table 2. Multiple linear regression analysis of handgrip strength and individual factors

	Men		Women	
	**β**	**P-value**	**β**	**P-value**
Age (years)	–0.261	<0.001	–0.302	<0.001
Body mass index (kg/m^2)	0.099	0.027	0.089	<0.001
C-reactive protein (mg/L)	0.012	0.773	–0.003	0.952
Creatine kinase (IU/L)	–0.004	0.919	0.076	0.067
Uric acid (μmol/L)	–0.029	0.515	–0.110	0.011
Cr/CysC ratio	0.112	0.013	0.170	0.001
Hemoglobin (g/L)	0.065	0.135	0.064	0.132
Albumin (g/L)	0.096	0.053	–0.015	0.736

β: standardized regression coefficient

Cr/CysC ratio = serum creatinine (mg/dL) / serum cystatin C (mg/L) × 10
